# Parsing rooms: the role of the PPA and RSC in perceiving object relations and spatial layout

**DOI:** 10.1007/s00429-019-01901-0

**Published:** 2019-07-17

**Authors:** Merim Bilalić, Tobias Lindig, Luca Turella

**Affiliations:** 1 Department of Psychology, University of Northumbria, Ellison Pl., Newcastle upon Tyne, NE1 8ST UK; 2 Department of Neuroradiology, University of Tübingen, Tübingen, Germany; 3 Center for Mind/Brain Sciences (CIMeC), University of Trento, Trento, Italy

**Keywords:** Scene perception, Parahippocampal place area (PPA), Retrosplenial cortex (RSC), Randomization, Object relations, Spatial layout, Visual search, Multivariate pattern analysis (MVPA)

## Abstract

The perception of a scene involves grasping the global space of the scene, usually called the spatial layout, as well as the objects in the scene and the relations between them. The main brain areas involved in scene perception, the parahippocampal place area (PPA) and retrosplenial cortex (RSC), are supposed to mostly support the processing of spatial layout. Here we manipulated the objects and their relations either by arranging objects within rooms in a common way or by scattering them randomly. The rooms were then varied for spatial layout by keeping or removing the walls of the room, a typical layout manipulation. We then combined a visual search paradigm, where participants actively search for an object within the room, with multivariate pattern analysis (MVPA). Both left and right PPA were sensitive to the layout properties, but the right PPA was also sensitive to the object relations even when the information about objects and their relations is used in the cross-categorization procedure on novel stimuli. The left and right RSC were sensitive to both spatial layout and object relations, but could only use the information about object relations for cross-categorization to novel stimuli. These effects were restricted to the PPA and RSC, as other control brain areas did not display the same pattern of results. Our results underline the importance of employing paradigms that require participants to explicitly retrieve domain-specific processes and indicate that objects and their relations are processed in the scene areas to a larger extent than previously assumed.

## Introduction

**Fig. 1:**
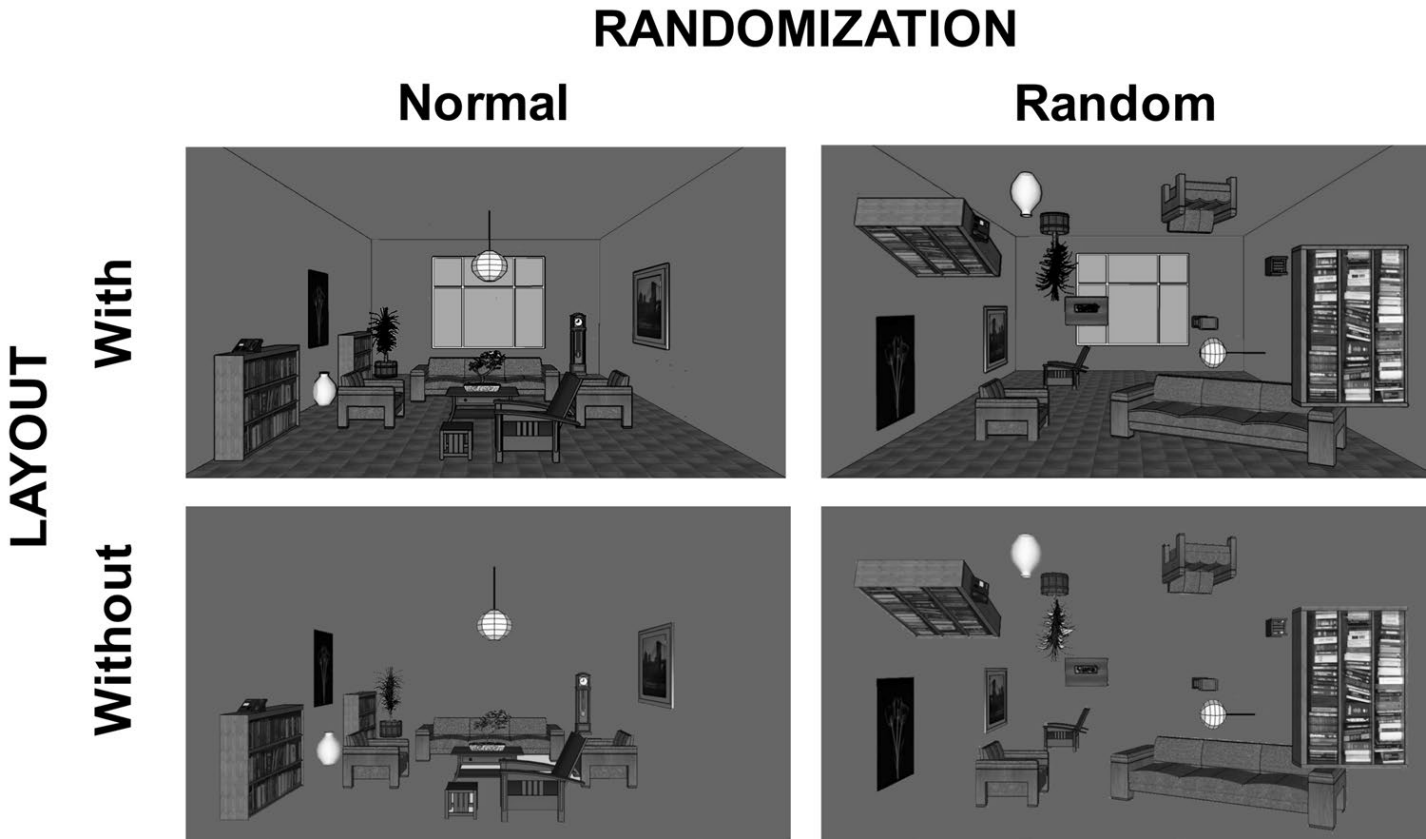
Stimuli and design. There were four room types organized around object relations and spatial layout factors. Arranging objects within a room in a normal or a random fashion manipulated the object relations. The layout factor featured rooms with and without background, in this case the walls. Crossing the two factors resulted in four room types: normal with layout, random with layout, normal without layout, and random without layout

Imagine that you are presented with a scene of a room, similar to the one depicted in Fig. 1. The chances are that you will quickly recognize that it is an indoor scene based on the space constrained by the walls. The objects in the scene, such as the table, sofa, chairs, and their arrangement will inevitably give away that the scene most likely depicts not only a room, but also a living room. This example illustrates that scenes can be recognized and categorized by both the global shape of the space, usually referred as the global or spatial layout (Oliva and Torralba 2001), and individual objects and relations between them (Biederman et al. 1974, 1982). The way in which the brain parses these two properties of scenes, especially the role of the scene-related areas, such as the parahippocampal place area (PPA) and the retrosplenial cortex (RSC) is currently unclear (Epstein 2008; Aminoff et al. 2013). One of the reasons for the ambiguity is that it is difficult to disentangle both scene factors. The other reason may lie in common paradigms that assume automatic activation of scene-related perceptual processes even when the participants were requested to passively observe stimuli. Here we present a novel paradigm that systematically manipulates both object and layout factors in a visual search task that requires active participation and guided attention. Using multivariate pattern analysis (MVPA), we demonstrate that the PPA and RSC differ in their sensitivity to the relations between objects in a room and to the room’s spatial layout.

Current theories of scene perception (Oliva and Torralba 2001; Torralba et al. 2006; Wolfe et al. 2011) assume that the initial quick impression based on the perception of the spatial layout is followed by slower, but possibly more informative perception of objects. For example, people can successfully categorize scenes as urban or natural after seeing them for as little as 25 ms (Greene and Oliva 2009; Rousselet et al. 2005; see also, Potter et al. 2014). People are also more likely to accurately recognize a scene when the objects in the scene are typical for that type of scene (e.g., priest in a church) than when they are not (Davenport and Potter 2004; e.g., football players in the church—Davenport 2007). It is fairly safe to say that both factors play important roles in scene perception (Malcolm et al. 2016).

The importance of spatial layout and objects in the scene is also evident in the way the human brain implements these two scene properties. The PPA, for example, seems to respond more strongly to spatial layout than to objects: empty rooms, which are characterized by walls and give rooms their typical layout, elicit similar activation levels to rooms filled with objects (Epstein and Kanwisher 1998). On the other hand, when objects usually found in rooms are presented separately, without the room to contextualize them, they do not activate the PPA nearly as much as empty rooms (Epstein and Kanwisher 1998; Epstein et al. 1999). More recent evidence (Kravitz et al. 2011a, b) demonstrates that the PPA is sensitive to the properties of spatial layout of scenes (open vs. closed), but cannot differentiate scenes based on the information provided by the objects in scenes.

The RSC also responds more strongly to scenes than to other stimuli, but is particularly important for spatial navigation (Epstein et al. 1999, 2007). The RSC will be more engaged than the PPA if people need to learn a route in an environment (Epstein 2008), navigate through a familiar place (Maguire et al. 1997), or recognize a scene as familiar (Ino et al. 2002). This also means that the RSC most likely needs to take into account the spatial layout as well as the individual objects in the scene to enable efficient navigation.

In contrast to the scene-related areas, the lateral occipital complex (LOC), which is generally thought to be responsible for processing object shape and category (Malach et al. 1995; Grill-Spector et al. 1999; Vinberg and Grill-Spector 2008), is more sensitive to individual objects in scenes than to layout properties of the scene (Epstein et al. 2003; Park et al. 2011; Harel et al. 2012).

The evidence for the importance of the spatial layout for PPA processing is unambiguous but the role of the PPA in the processing of objects in the scenes is currently unclear. On the one hand, object properties such as size (Konkle and Oliva 2012; Troiani et al. 2014), spatial distance (Cate et al. 2011; Amit et al. 2012), and space definition (Mullally and Maguire 2011) are also represented in the PPA. This may indicate that the PPA processes object features, but these features also seem to directly influence spatial properties of scenes. It is reasonable to assume that the spatial layout would be influenced by these features too. On the other hand, there is evidence that the PPA, and the parahippocampal gyrus in general, may be involved in parsing functional and spatial relations of objects in addition to scene layout. Bar and colleagues (Aminoff et al. 2007; Bar et al. 2008; Cheung and Bar 2012, 2014) found more PPA activity when participants perceived isolated objects with strong association (e.g., an oven, which is associated with a typical context of kitchen) than objects with weak context (e.g., a camera, which is found in various different situations). This contextual effect, however, seems to be present only with longer exposure (Epstein and Ward 2010).

Our own studies on chess expertise (Bilalić et al. 2010, 2012) highlight the importance of the PPA and RSC for the processing of spatial relations between objects. Chessboards, with layout (board frame) and objects (chess pieces) essentially present scenes and have activated the PPA and RSC in our studies, particularly in chess experts. However, when the objects were randomly scattered on the chessboard, experts activated the PPA and RSC less than when they perceived normal configuration of chess objects. Random configurations of objects do not make sense for experts, as they cannot process the functional and spatial relations between objects. In contrast, the PPA and RSC in novice chess players, who do not possess this domain-specific knowledge about relations between chess objects, were not sensitive to the randomization of chess objects. Other studies using the same randomization paradigm, but different domain-related tasks, which required domain-specific activities from participants, reproduced the same pattern of results in experts’ PPA and partly in RSC (Campitelli et al. 2005, 2007; Bartlett et al. 2013).

There have been attempts to disentangle the role of spatial layout from that of functional relations between objects in the scene-related areas, such as the PPA and RSC, but none of them manipulated the functional and spatial factors using the randomization paradigm. Both of the studies mentioned above (Park et al. 2011; Kravitz et al. 2011a) employed scenes that manipulated content (man-made/urban and natural), but did not directly investigate natural relations between the content elements. Harel et al. (2012) systematically manipulated layout and content, but due to experimental control restrictions only employed one object at a time. Similarly, in the studies that used rooms with furniture (Epstein and Kanwisher 1998; Henderson et al. 2006; Harel et al. 2012; Kamps et al. 2016), usually only one aspect of a room, a single wall, with only a few objects placed against it, was used.

The manipulation of spatial and functional relations by randomization of objects has often been used as a key paradigm in a number of fields, such as memory (Mandler and Parker 1976; Mandler and Ritchey 1977; Tulving 1962, 1983), perception (Biederman et al. 1973; Mandler and Johnson 1976), and expertise (Chase and Simon 1973; Ericsson and Lehmann 1996; Vicente and Wang 1998). Recently, Võ and Wolfe (2013a, b) showed that spatial and functional information is one of the main guiding features in the perception of naturalistic scenes, such as rooms, by employing a variation of the randomization paradigm. The randomization paradigm may, therefore, be a way to investigate the roles of spatial layout and object functions in the perception of scenes and how they are processed in the PPA and RSC.

Randomly scattering objects within a living room disturbs typical spatial and function relations between those objects. The placement of objects within a room is often a direct consequence of their function—we are hardly likely to find a TV set on ceiling, as it would be too uncomfortable to watch. At the same time, the randomization disturbs objects but leaves the key factor of the spatial layout, the room walls, intact. The randomization may, therefore, present an important test of the brain areas responsible for scene perception. If the PPA is mostly related to spatial layout properties, then the manipulation involving spatial relations between objects within a scene should not have a big effect. In contrast, if the PPA does indeed play a role in contextual perception, as some claim (Bar 2004; Aminoff et al. 2007, 2013; Bar et al. 2008; Bilalić et al. 2010, 2012; Cheung and Bar 2012), then the randomization of objects should be reflected in the PPA activation even when there is no spatial layout in the scene. Similarly, both spatial layout and object placement within the scene may be important for navigation. The question remains, however, whether both factors will activate the RSC, and to what extent.

Here we tested these hypotheses by presenting scenes of living rooms where objects were normally allocated, i.e., in their natural positions, and where they were scattered randomly around the room. We also manipulated the layout factor by presenting the rooms with and without walls (see Fig. 1 for an illustration of stimuli). Importantly, participants were asked to actively manipulate the stimuli, that is, search for a particular object within the room. This is in contrast to most previous research on scene perception, in which people were only required to passively observe presented stimuli^1^ (Epstein and Kanwisher 1998; Park et al. 2011, 2015; Kravitz et al. 2011a; Harel et al. 2012; Kamps et al. 2016). The underlying assumption is that the main processes in scene perception are automatically elicited (for a review, see Harel 2015a). There are numerous behavioral studies that demonstrate that for simple categorizations (e.g., urban/landscape, animal/non-animal) explicit attentional resources may not be necessary (Rousselet et al. 2005; Greene and Oliva 2009; Poncet et al. 2012). However, there are a growing number of behavioral studies showing the necessity of attention for certain cognitive processes in scene perception (Potter and Fox 2009; Cohen et al. 2011). While the debate about the role of attention in scene perception is highly topical in behavioral research (Gronau and Izoutcheev 2017; Hansen et al. 2018), the fMRI research still predominantly employs a passive paradigm which presumably relies on automatic activation.

Here we use active search and explicit attentional direction, which is in line with our previous experiments using the randomization paradigm (Campitelli et al. 2005, 2007; Bilalić et al. 2010, 2012; see also, Wan et al. 2011). In these experiments, it was necessary to explicitly perceive spatial relations between the objects within a scene to execute the task. The randomization paradigm in combination with active search produced the randomization effects in the PPA, RSC, and even in the FFA (Bilalić et al. 2011). Other recent research on visual expertise (Harel et al. 2010; McGugin et al. 2014b; Wong et al. 2014) demonstrates that top-down processes, such as retrieval of knowledge or allocation of attention to different aspects of stimuli, result in different patterns of brain activation from situations when these processes are not necessary (for reviews, see Harel 2015a, b; Bilalić 2017).

We, therefore, expect that the randomization paradigm combined with top-down modulation will enable us to uncover whether the scene brain areas are indeed sensitive to functional relations of objects within scenes. A number of older studies using the classical univariate analysis could not establish a link between object relations and scene areas (Epstein and Kanwisher 1998; Epstein et al. 1999, 2008; Downing 2005; Kim et al. 2011). The more sensitive MVPA, which we apply here, could uncover differentiation between categories even when there are no differences between categories in univariate analysis (Haxby et al. 2014). More importantly, MVPA can also establish whether it is possible to generalize stimuli based on object function without regard for other scene properties such as spatial layout of images (e.g., cross-generalization).

It is essential to demonstrate the sensitivity of the scene-related areas to the randomization of objects, but it is also crucial to demonstrate that other similar brain areas are not engaged in the same processes. For this purpose, we have chosen two control areas, the aforementioned LOC and the fusiform face area (FFA). The LOC is particularly suitable because it has recently emerged that it not only processes basic object properties, but also relations between objects (MacEvoy and Epstein 2011; Kim et al. 2011; Preston et al. 2013). The FFA, which is in the vicinity of the PPA, and as such is an anatomically suitable control area, is related to face processing (Kanwisher et al. 1997; Kanwisher and Yovel 2006) but also to holistic processing in expert domains (Gauthier et al. 1999, 2000; Bilalić et al. 2011, 2016; McGugin et al. 2012, 2014a; Bilalić 2016). A key component of holistic processing is the spatial and functional relations between individual elements of the stimuli (Tanaka and Farah 1993; Cheung et al. 2008; Richler et al. 2012), which are precisely the factor we manipulate with the randomization paradigm. Should the perception of spatial layout and object relations in the scene not be confined to the PPA and RSC, the LOC and FFA are good candidate brain areas for finding these effects.

## Method

### Participants

There were 15 participants (3 female, *M* = 29.1 ± SD = 5). All participants were right-handed, had normal or normal-to-corrected vision, and no known neurological issues. The informed consent was obtained in line with the Institutional Review Board of the Ethics Committee of Tübingen University.

### Tasks, stimuli and design

The room stimuli were generated using Google SketchUp software (http://www.sketchup.com) to look like common living rooms with typical objects such as chairs, tables, sofas, TV sets, drawers, bookshelves, plants, and lamps. We initially generated 60 rooms, each containing between 14 and 18 objects. The rooms were pilot-tested not only for the current task (search for an object within the room), but also for general recognition of the room, as well as for how readily discernible the main elements (e.g., walls^2^ and objects) were. In the end, we used 51 of these rooms in the experiment. Each of the 51 generated rooms featured different walls and different objects (e.g., different types of sofas). Each of the stimuli had four versions that systematically varied the layout and objects in rooms, which produced altogether 204 room stimuli. There were rooms that contained the walls as background and those that did not (factor layout). There were rooms that featured normal arrangement of objects and those rooms where the objects were randomly scattered around the room (factor randomization). These two factors produced a 2 × 2 design with four different types of rooms (see Fig. 1). We randomized the objects within rooms by dividing each room into 30 parts (6 horizontally × 5 vertically) and randomly generating where each object would be placed. In this way, we have the same objects in normal and random rooms, differing only in their arrangements. The objects in the random rooms were inevitably placed in uncommon spaces and we decided to use the walls in all instances for placement. This resulted in objects hanging from the wall upside down in unusual orientations. This, in our opinion, is less unnatural than objects presented in their typical orientation floating in space (see, Biederman 1981).

The dimensions of the whole stimulus were 400 × 220 pixels. The stimuli were projected onto a screen above the heads of the participants via a video projector in the adjacent room. Participants saw the stimuli through a mirror mounted on the head coil. The physical dimensions of the stimulus were 336 × 184 mm. The setup resulted in a visual field of 13.8° for the whole stimulus. The task was to indicate if a TV set was present in the room by pressing the left button of an MRI-compatible answer-pad for present (yes) or the right button for absent (no). The pad was held in participants’ right hand. The task is essentially a visual search (yes–no) task with factors layout (wall/no-wall) and randomization (normal/random) manipulated.

There were three experimental runs. A single run started with a baseline (gray screen with a cross in the middle) lasting 10 s and ended with the same baseline lasting 12 s. Between the baselines were presented 17 trials of each condition making altogether 68 trials (4 conditions × 17 trials). A single trial lasted for 3 s and was followed by a baseline lasting between 2 s and 9 s (with most baselines falling between 3 and 4 s). A black cross presented centrally in the baseline would turn red half a second before the trial was presented. That way the participant was warned about the upcoming trial. The trials were counterbalanced over four conditions for each participant separately using Optseq program (Dale 1999).

### Localizer experiment

All participants were initially presented with a localizer run that featured the following stimuli categories: pictures of room interiors as place category (taken from the Internet), pictures of faces (taken from Leube et al. 2001), pictures of man-made objects (taken from Brodeur et al. 2010), pictures of normal chess positions, and pictures of isolated chess objects. In the localizer (see below), only the relevant stimuli categories were used, i.e., compared. All stimuli were converted to black and white, spanned 250 × 250 pixels and had physical dimensions of 210 × 210 mm, which resulted in a visual field of 12.8°.

The localizer was a block design similar to the one used in the first experiment. The blocks lasted for 12 s and contained 6 stimuli presented for 1.75 s followed by a stimulus mask for 0.25 s. There were 8 blocks of each condition. The localizer started with a baseline (gray screen with a cross in the middle) lasting 12 s and finished with the same baseline lasting 18 s. There was always another baseline lasting 18 s after each of the eight cycles of 5 blocks (one for each condition). The presentation of blocks was counterbalanced separately for each participant. The task was to spot direct repetition (1-back task). There was one repetition in each of the blocks.

### Imaging data acquisition

We acquired fMRI data using a 3T scanner (Siemens Trio) with a 12-channel head coil at the fMRI center in Tübingen, Germany. We covered the whole brain using a standard echo-planar-imaging sequence with the following parameters: [RT] = 2.5 s; [FOV] = 192 × 192; [ET] = 35 ms; matrix size = 64 × 64, 36 slices with thickness of 3.2 mm + 0.8 mm gap resulting in voxels with the resolution of 3 × 3×4 mm^3^. Anatomical images covering whole brain with 176 sagittal slices were obtained after the functional runs using an MP-RAGE sequence with a voxel resolution of 1 × 1×1 mm^3^ (TR = 2.3 s, TI = 1.1 s, TE = 2.92 ms).

### Functional univariate MRI data analysis

The preprocessing was done with SPM8 and involved spatial realignment to the mean image including unwarping and co-registration of the anatomical image to the mean EPI. We did not perform segmentation, normalization or spatial smoothing procedures because we wanted to use original unstandardized data for the univariate analysis (and later for MVPA). For the univariate analysis, we modeled the trials explicitly for the duration (3 s) as a single regressor for each stimulus type, while the baseline was modeled implicitly in a general linear model [hemodynamic activation modeling relied upon a canonical response function, AR(1) and a 128 Hz high-pass filter]. We also added six movement parameters in the GLM to account for the variance introduced through head motion. All preprocessing and analyses were done by SPM8.

**Fig. 2:**
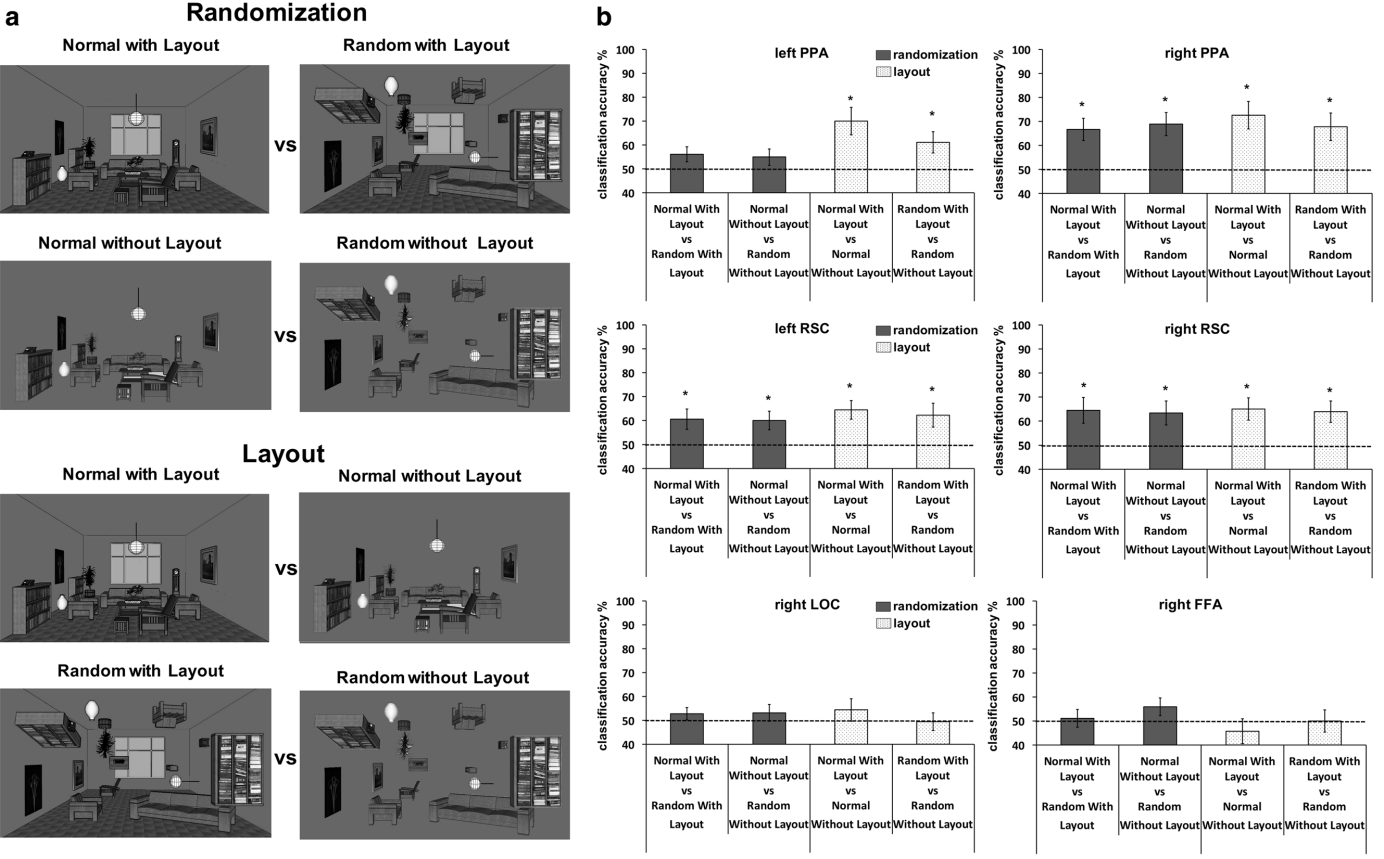
MVPA results. **a** MVPA procedure. **b** Classification accuracy presented in percentage of correctly classified instances (50% is a chance level—see the dotted line) of the four binary comparisons for the left and right PPA (top panel), the left and right RSC (middle panel), and the right LOC and right FFA (bottom panel). Error bars represent standard error of the mean (SEM). *p < 0.0125 (corrected for multiple comparisons); ^**+**^*p* < 0.05

The MarsBaR SPM Toolbox (Brett et al. 2002) was used to extract the percent signal change (relative to baseline) for each participant in each ROI depending on the room type. These activation levels were then plotted (Fig. 2) and analysed using *F* and *t* tests.

### Multivariate pattern analysis (MVPA)

The MVPA analyses used the same preprocessing procedure as the univariate analysis (see above). Unlike the univariate analysis, where we modeled all trials of the same type together, for the MVPA we modeled each single trial. We controlled for RT on different trials by calculating mean RTs across the four room types and subtracted the individual trial in the run from the corresponding mean. The obtained values were used as an additional variable at the individual level to control for different reaction time between different room types (Todd et al. 2013).

**Fig. 3:**
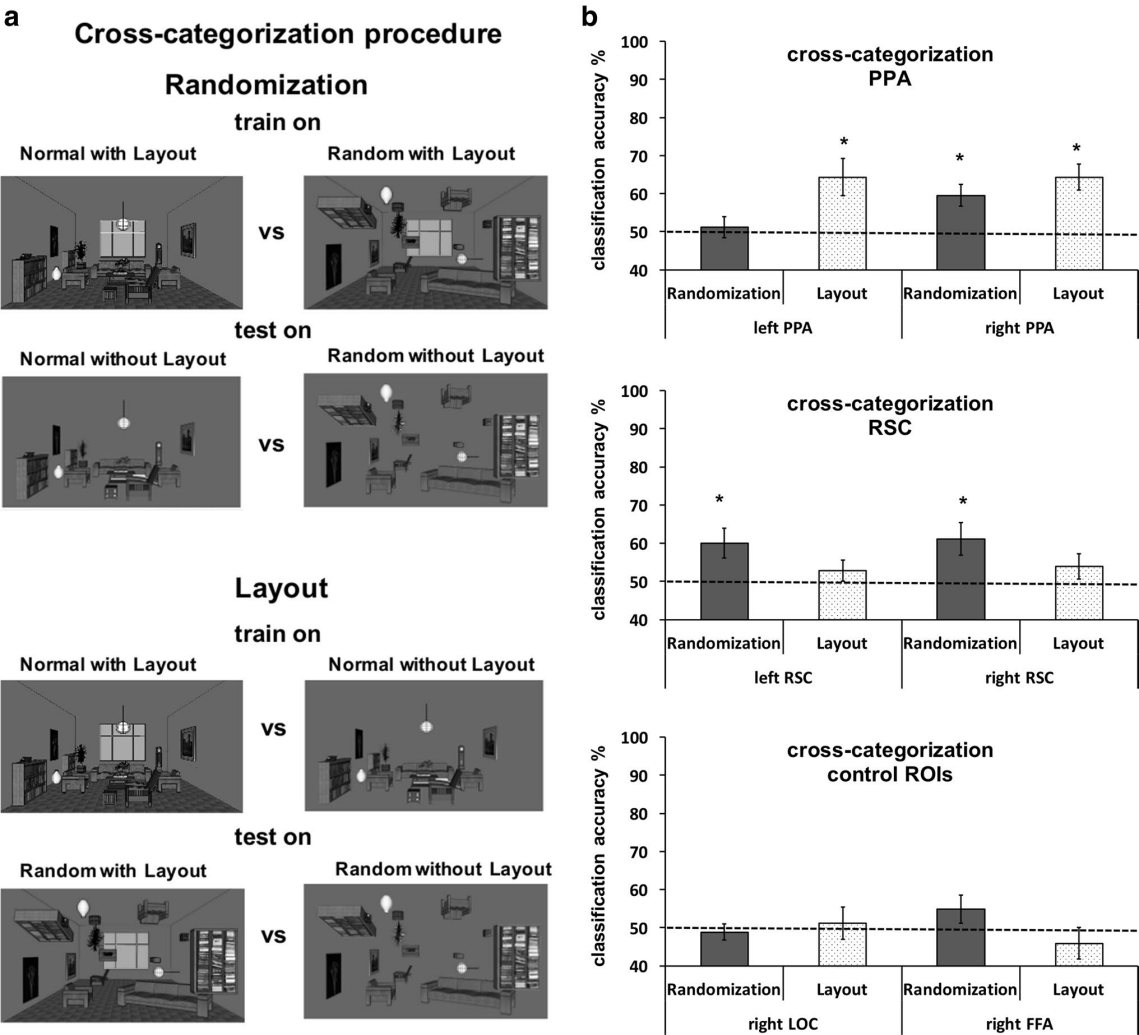
MVPA cross-categorization results. **a** Cross-categorization procedure. **b** Cross-categorization accuracy presented in percentage of correctly classified instances (50% is a chance level—see the dotted line) of the two binary comparisons for the left and right PPA (top panel), for the left and right RSC (middle panel), and for the right LOC and right FFA (bottom panel). Error bars represent SEM. *p < 0.025 (corrected for multiple comparisons)

We performed the MVPA analyses using the Decoding Toolbox (Hebart et al. 2015). The toolbox uses support-vector-machine (SVM) method of multivariate pattern analysis (MVPA) to see if the localized PPAs (see below) differentiate randomization and layout properties of scenes. Our comparisons were binary SVM classifications and centered around the two factors (see Fig. 3a). For the randomization factor we compared normal rooms versus random rooms when they both had layout (first randomization binary classification) and when they both were without layout (second randomization binary classification). The layout factor also involved two binary classifications, but this time we compared normal rooms with layout versus the normal rooms without layout (first layout binary classification) and random rooms with layout versus random rooms without layout (second layout binary classification). For all classifications, a linear SVM with standard cost parameter, *c* = 1, as implemented in the LIBSVM 3.0 library (Chang and Lin 2011) was used. The classification was based on the *β* values previously obtained by the GLM and all voxels in a single ROI. We employed a leave-one-trial-out cross validation method (e.g., Sterzer et al. 2008) where the dataset was divided into (1) a training set of *N* pattern vectors (vector length = number of voxels) and, (2) a validation set of two pattern vectors, one from each stimulus type. We then scaled the *β* in all training sets (0-1) as well as in validation sets. The SVM classifier was iteratively trained on the training datasets (*N*) and then tested on an independent validation dataset. The individual trials were used as basic units for comparison. These training and validating procedures were repeated 100 times. Percentage of successful categorization of tests items based on the previous independent training data was obtained for each comparison and for each participant. At the group level, we tested with one-sample, one-sided *t* tests if the average classification accuracy among the participants for the binary comparison in question was significantly greater than the chance level (50%). Given that there were four binary comparisons, the significance level was set at *p* = 0.0125 (the standard 0.05 significance level divided by the number of comparisons, 4).

### Cross-categorization MVPA

We also performed a stronger test of the influence of randomization and layout factors in PPA and RSC. For the randomization factor, we first trained the binary classifier on all possible normal versus random rooms with layout comparisons and validated on the same normal versus random comparisons, but this time on rooms without layout (see Fig. 3a). This way we always use the randomization comparison (normal vs. random) but train on rooms with layout and test on different stimuli—rooms without layout. We also checked the other direction in the cross-categorization procedure (random vs normal instead of the described normal vs random). The presented results were then the averaged for both direction (both direction produced almost identical classification accuracies, the difference being within 1%). For the layout factor, we trained on normal rooms with layout versus normal rooms without layout and validated on random rooms with layout versus random rooms without layout (again, we also tested for the other direction—normal without layout vs normal with layout). Here the manipulation was the layout comparison (with vs. without) while the randomization factor was constant and was used as training and testing sets. If any of the factors play a role in the PPA and RSC’s functioning, then the PPA and RSC should be sensitive even if the learned patterns are tested on different stimuli.

The cross-categorization procedures which start with the comparison between random and normal (instead of the normal vs random) were also conducted. The results were within 1% of the presented results. The group level analysis was the same as in the previous MVPA analysis, but the significance level was set at *p* = 0.025 because there were only two comparisons.

### Localizer analysis

To isolate the PPA, we modeled the blocks for each condition in the localizer run while the baseline was implicitly modeled in a GLM. We then compared the blocks with places (interior of rooms) with only the blocks with faces (blocks with other stimuli were not compared). The voxels in the vicinity of the collateral sulcus that survived the *p* < 0.0001 (uncorrected) threshold were then taken as the PPA ROI. In three of the participants we used a less stringent threshold (*p* < 0.001) to identify the bilateral PPAs. The left PPA was on average, *M *=665 ± SD=211 mm^3^, the right PPA, 782 ± 284 mm^3^. The same procedure was carried out for the RSC, only here we looked at the activated voxels near the posterior cingulate and parieto-occipital sulcus. In two participants, we employed a less stringent threshold (*p* < 0.0001) than in others (*p* < 0.001). The left RSC was on average, *M *=430 ± SD=174 mm^3^, the right RSC, 521 ± 197 mm^3^.

For the sake of completeness, we also isolated another scene region, the occipital place area (OPA). Our paradigm is not intended to test the OPA properties and we, therefore, report its results in the Appendix. The OPA was isolated by taking the voxels around the transverse occipital sulcus (OTS) which were activated at a level greater than *p* < 0.0001 when we compared the blocks of places with the blocks of faces. The left OPA was, on average, *M *=649 ± SD=241 mm^3^, the right OPA, 738 ± 203 mm^3^.

We also used the reverse contrast (Face vs. Place) to identify the right fusiform face area (Kanwisher et al. 1997) that we used as a control ROI. The voxels in the right fusiform gyrus that were still activated after applying the *p* < 0.0001 (uncorrected) threshold were then taken as the right FFA ROI. The right FFA was on average 672 ± 138 mm^3^.

The LOC was identified by comparing man-made objects with the baseline and looking at the activated voxels around the lateral occipital area and the posterior fusiform gyrus. The voxels that survived the *p* < 0.0001 threshold were chosen in the ROI. The right LOC was on average 684 ± 276 mm^3^.

Similar to the OPA, the visual cortex, V1, was also included in the control regions, although it is unclear what kind of pattern of results one would expect in V1. Unlike the previous ROI, the V1 was not obtained functionally from individual ROIs of participants because we did not have a functional localizer for V1. We used the anatomical V1 (Amunts et al. 2000) provided in the Anatomy Toolbox (Eickhoff et al. 2005). The ROI was then used in combination with normalized functional scans. As with the other control ROIs, we used the right V1. The results of V1 are presented in the Appendix.

### Anterior–posterior PPA

Recent research (Baldassano et al. 2013, 2016; Aminoff and Tarr 2015) suggests that the PPA may not be a homogeneous area as previously believed. The anterior PPA seem to be connected to OPA and involved in object processing, whereas the posterior PPA parses layout properties of scenes (Baldassano et al. 2013; Aminoff and Tarr 2015). Here we checked the activation pattern in each voxel of the individual ROIs for both MVPA and cross-categorization MVPA. We exploited the property of the PPA where its voxels follow anterior–posterior axis across the *y* coordinate with higher negative numbers indicating more posterior voxels. We calculated Pearson’s correlation between a voxel’s *y* value and MVPA classification success for each participant and each binary MVPA comparisons. The individual correlation coefficients were then Fisher* z*-transformed (Fisher 1921) and averaged for each of the four binary comparisons. The positive correlation indicated more success for the posterior voxels, while the negative correlations denoted better success of the anterior part of the PPA.

## Results

### Behavior analysis

We asked participants to actively seek for an object (TV set) in rooms. The rooms were again categorized based on the randomization and layout factors and could feature normal and random arrangements of objects, as well as be with and without layout. We know that common relations between objects enable efficient search (Biederman et al. 1982; Bilalić et al. 2010, 2012; Võ and Wolfe 2013a, b) and we expected the participants to be particularly fast in normal rooms and slower in random ones. This indeed happened. The participants were more accurate (83%) and faster (1.62 s on average) in normal rooms with layout than in random rooms with layout (80% and 1.82 s for accuracy and speed, respectively). The presence of the layout did not alter the pattern. The participants were still more accurate and faster in normal rooms without layout (79%, 1.56 s) than in random rooms without layout (77%, 1.73 s). When we run a 2 × 2 analysis of variance (ANOVA) with randomization and layout factors for accuracy and reaction time separately, we found both main factors significant or almost significant [randomization *F*(1, 14) = 16.1, *p* < 0.001; layout *F*(1, 14) = 4.4, *p* = 0.056 for accuracy, and randomization *F*(1, 14) = 55.8, *p* < 0.001; layout *F*(1, 14) = 28.1, *p* < 0.001 for RT] and no interaction between them [*F*(1, 14) = 0.1, ns for accuracy, and *F*(1, 14) = 1.2, *p* = 0.29 for RT].

### Univariate fMRI analysis

**Fig. 4:**
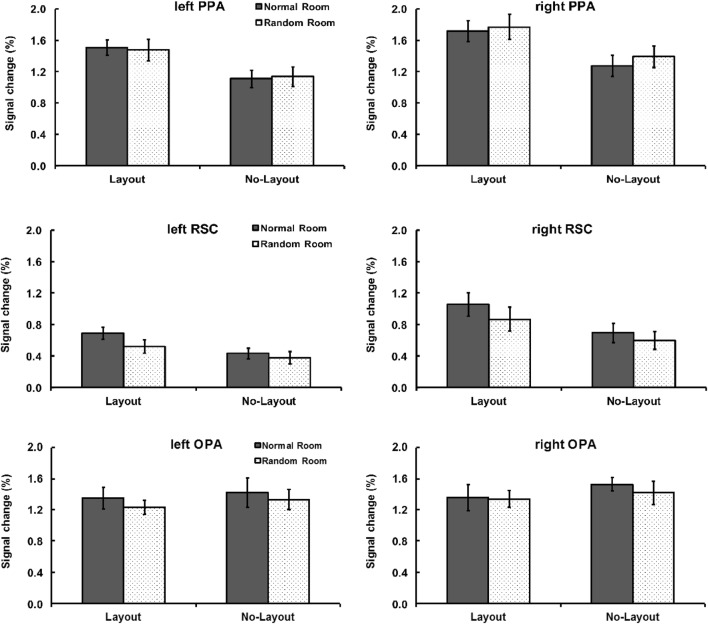
Univariate fMRI results of scene-related and control regions. Activation levels (% signal change from the baseline) for normal (black) and random (white) rooms with or without layout for the left and right PPA (top panel), left and right RSC (middle panel), and left and right OPA (bottom panel)

**Fig. 5:**
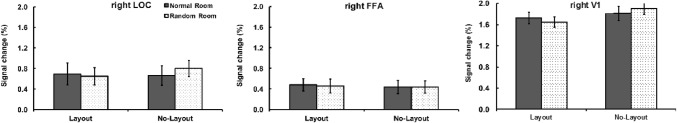
Univariate fMRI results of control areas. Activation levels (% signal change from the baseline) for normal (black) and random (white) rooms with or without layout for the right LOC (left panel), right FFA (middle panel), and right V1 (right panel)

Detailed analyses are provided in the Appendix (Fig. 4). Here we merely summarize the results. We confirmed that the PPA could differentiate between the rooms with different layouts, but that the randomization of the objects within the room had no significant effect. In contrast, the RSC was sensitive to both factors, while the OPA was not reacting significantly different to the four types of rooms. There were also no main effects of randomization or layout in the control ROIs, right FFA, LOC, and V1 (see Appendix, Fig. 5).

### Multivariate fMRI analysis

The univariate analysis showed that the PPA responded only to the layout, whereas the RSC was modulated by both layout and randomization. Here we present the more sensitive MVPA, which should have a better chance of capturing the PPA response to the randomization if there is indeed any effect. Given that MVPA is also sensitive to the effort as indexed by reaction time (Todd et al. 2013), the ROI activations were controlled for the RT as indicated in the method section. The *β*s used as the input for the MVPA were, therefore, corrected for the RT.

Figure 2a depicts the binary categorizations we used. Figure 2b shows the results of these binary categorization tests. The left PPA did not discriminate between different randomization categories—normal rooms and random rooms with layout [*t*(14) = 1.98, *p *= 0.034] and normal and random rooms without layout [*t*(14) = 1.46, *p *= 0.08]. The left PPA did, however, differentiate between rooms with and without layout—*t*(14) = 3.52, *p *= 0.002 for normal with vs. normal without layout, and *t*(14) = 2.48, *p *= 0.014 for random with vs. random without layout rooms. In contrast, the right PPA was sensitive to both randomization and layout factors. It differentiated between normal and random rooms with layout [*t*(14) = 3.62, *p *= 0.001], as well as normal and random rooms without layout [*t*(14) = 3.9, *p *= 0.001]. It also distinguished normal rooms with and normal rooms without layout [*t*(14) = 3.98, *p *= 0.001] just as it differentiated between random rooms with and random rooms without layout [*t*(14) = 3.09, *p *= 0.004]. The direct comparison between the left and right PPA in a three-way ANOVA with factors layout (with-without), randomization (normal-random), and side (left–right) showed that the right PPA was significantly more successful than the left PPA [main effect side: *F*(1, 14) = 15.3, *p* = 0.002]. The difference was driven by the randomization factor [*F*(1, 14) = 11.9, *p* = 0.004] rather than by the layout factor [*F*(1, 14) = 1.6, *p* = 0.23].

It is important to emphasize that these analyses (and the others reported later) were not a result of different reaction time or accuracy for different room types. When we include only the accurate trials in the analyses, we get essentially the same classification accuracies. Similarly, there were no significant correlations between single subject classification accuracy of the binary comparisons and the difference in the reaction time of the same room types that were compared: normal with layout vs. random with layout [*r*(14) = 0.21 for left, and *r*(14) = 0.09 for right PPA], normal without layout vs. random without layout [*r*(14) = 0.29 for left, and *r*(14) = 0.36 for right PPA], normal with layout vs. normal without layout [*r*(14) = 0.04 for left, and *r*(14) = 0.29 for right PPA], and random with layout vs. random without layout [*r*(14) = 0.28 for left, and *r*(14) = 0.12 for right PPA].

Following recent research (Baldassano et al. 2013), which suggests that the PPA was not a homogeneous region, we check the success rates of all individual PPA voxels. The Fisher’s *z*-transformed correlation between the *y* coordinate, which indicated the anterior–posterior axis in the PPA, and the MVPA success was positive for the randomization comparisons (Mcorr = 0.05 ± SE = 0.09 and 0.06 ± 0.06 for normal vs. random rooms with layout, and normal vs. random rooms without room, respectively) and negative for the layout factor (Mcorr = − 0.08 ± SE = 0.06 and − 0.14 ± 0.05 for normal with layout vs. normal without layout, and random with layout vs. random without layout, respectively) in the left PPA. The same pattern of results was found in the right PPA: the more anterior right PPA voxels were better at differentiating between normal and random rooms (Mcorr = 0.04 ± SE = 0.06 and 0.19 ± 0.07 for normal vs. random rooms with layout, and normal vs. random rooms without room, respectively), whereas the more posterior right PPA voxels distinguished better between rooms with and without layout (Mcorr = − 0.08 ± SE = 0.07 and − 0.11 ± 0.08 for normal with layout vs. normal without layout, and random with layout vs. random without layout, respectively).^3^

Both left and right RSC were able to differentiate between the four comparisons (Fig. 2), which may not be surprising given that the univariate analysis had already produced effects (see Fig. 4). The left RSC successfully distinguished between normal and random rooms with layout [*t*(14) = 2.53, *p *= 0.012], normal and random rooms without layout [*t*(14) = 2.55, *p *= 0.012], as well as normal rooms with and normal rooms without [*t*(14) = 3.67, *p *= 0.002], and random rooms with layout and random rooms without layout [*t*(14) = 2.44, *p *= 0.012]. The same pattern of results was found in the right RSC, which could also distinguish between different room types [normal and random rooms with layout—*t*(14) = 2.69, *p *= 0.009; normal and random rooms without layout—*t*(14) = 2.71, *p *= 0.008]; normal rooms with and normal rooms without—*t*(14) = 3.25, *p *= 0.003; random rooms with layout and random rooms without layout—*t*(14) = 3.19, *p *= 0.003. Unlike with the PPA, there was no significant difference between the right and left RSC (All Fs > 1). The MVPA accuracy values of the RSCs were also not significantly correlated with the reaction time participants needed to find the object in the rooms (all *r *< 0.31).

The MVPA analysis for the left OPA yielded a significant result in the comparison between normal and random rooms without layout. However, all other comparisons, including those for the right OPA, were not significantly reliable (see Appendix, Fig. 5).

The control areas, the right FFA and LOC, were not as successful in differentiating between the four binary comparisons (all comparisons, *p* > 0.10). The right V1 could only differentiate between normal and random rooms with layout, but all other comparisons were not significant (see the Appendix, Fig. 8).

### Multivariate cross-categorization fMRI analysis

The cross-categorization procedure is presented in Fig. 3a. Figure 3b shows the results of the cross-categorization procedure. The left PPA was only sensitive to layout [*t*(14) = 3.17, *p *= 0.004] and not to randomization of objects [*t*(14) = 0.64, ns]. The right PPA, however, responded to both randomization [*t*(14) = 3.67, *p *= 0.002] and layout [*t*(14) = 4.52, *p *< 0.001]. The RSC displayed a different pattern of results. Both left and right RSC were able to cross-categorize rooms successfully based on the randomization [*t*(14) = 2.53, *p *= 0.012 and *t*(14) = 2.64, *p *= 0.008, for the left and rights RSC, respectively], but the same was not possible based on the layout [*t*(14) = 1.01, *p *= 0.16 and *t*(14) = 1.16, *p *= 0.13, for the left and right RSC, respectively]. The differences between the PPA and RSC are also evident as the right PPA was significantly more successful than its left counterpart in the randomization [paired *t* test, *t*(14) = 2.2, *p* = 0.042] but not in the layout (*t *< 1, ns). There were no differences in the success rate between the right and left RSC either in the randomization or layout (*t *< 1, ns).

**Fig. 6:**
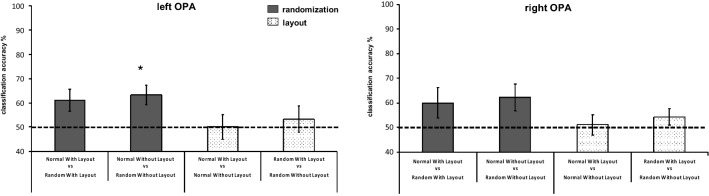
MVPA results. Classification accuracy presented in percentage of correctly classified instances (50% is a chance level—see the dotted line) of the four binary comparisons for the left and right OPA *p < 0.0125 (corrected for multiple comparisons); ^**+**^*p* < 0.05

**Fig. 7:**
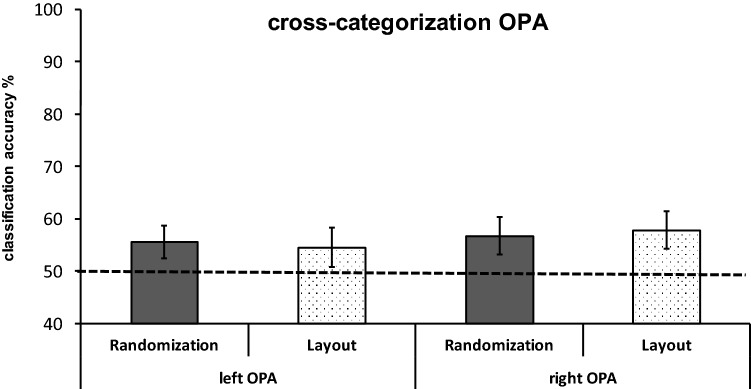
MVPA cross-categorization results. Cross-categorization accuracy presented in percentage of correctly classified instances (50% is a chance level—see the dotted line) of the two binary comparisons for the left and right OPA. *p < 0.025 (corrected for multiple comparisons)

**Fig. 8:**
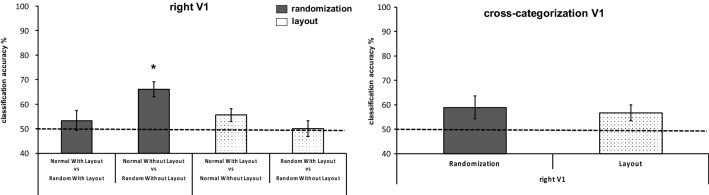
MVPA analyses for V1. Classification accuracy presented in percentage of correctly classified instances (50% is a chance level—see the dotted line) of the four binary comparisons for the right V1 (left panel). Cross-categorization accuracy presented in percentage of correctly classified instances for right V1 (right panel). *p < 0.0125 (corrected for multiple comparisons)

The left and right OPA could not reliably cross-categorize rooms along the randomization or layout factor (see Appendix, Fig. 6). The same results were also found for the control ROIs, the right FFA, the right LOC, and V1-none of them could cross-categorize the rooms across either of the factors (all *p* > 0.20—see Figs. 3, and 7, 8 in the Appendix).

## Discussion

Our main goal was to test whether the scene-related brain regions, the PPA and RSC are sensitive to the relations between objects, as well as to the layout of the scene. We manipulated the common relations between objects within a room employing the randomization paradigm, while the layout factor was controlled by presenting those rooms with and without the typical walls (see Fig. 1). In a visual search paradigm where the participants had to actively search for an object in rooms, the univariate analysis demonstrated that both the left and right PPA were sensitive only to the layout properties and not to the object randomization (see Appendix). The left and right RSC, in contrast, were sensitive to both layout and object factors, which was already apparent in the univariate analysis. The MVPA confirmed that both left and right RSC can differentiate between different rooms taking into account both layout and objects (see Fig. 3). The MVPA also demonstrated that the right PPA can differentiate between the rooms using the information about relations of the objects in the rooms. The final cross-categorization MVPA analysis confirmed that both the left and right PPA can indeed differentiate between the rooms based on the layout properties irrespective of the objects in the rooms, but that only the right PPA can differentiate rooms using on the relations of objects in the room irrespective of the layout. In contrast, the left and right RSC could only transfer the information about the relations between objects, that is the randomization factor, to categorization of different stimuli. The layout factor was not strong enough in the RSC to be transferred for differentiation of a new class of stimuli.

Despite the evidence that the right PPA is sensitive to the relations between objects within scenes, it also apparent that both the left and right PPA respond to the layout properties of scenes. Even cross-categorization tests, where the properties of one type of the binary comparison are tested on another binary comparison, indicate that both PPAs are successful in differentiating between rooms with and without layout irrespective of their object arrangements. These results corroborate and extend previous findings on the role of spatial layout in PPA (Epstein and Kanwisher 1998; Epstein 2008; Park et al. 2011; Kravitz et al. 2011a). That is, however, only one side of the story, because the right PPA could not only differentiate based on the relation between objects in the scene, but also use this information to differentiate between new stimuli with the same relations. These results indicate that the PPA’s function is related to both object and layout factors in scenes. This possibility may go a long way towards explaining seemingly contradictory pattern of results that we currently have when it comes to the PPA’s function, as there is ample evidence that the PPA is sensitive to layout manipulation (Epstein and Kanwisher 1998; Park et al. 2011; Kravitz et al. 2011a), but also to objects and their relations (Aminoff et al. 2007; Bilalić et al. 2010, 2012; Harel et al. 2012).

That the PPA may respond to both layout and object relations is supported by anatomical evidence from monkey studies. Unlike other scene-relevant areas such as the LOC, the PPA receives input from both ventral parts of V4 (Ungerleider et al. 2008) and the dorsal visual pathway through the parietomedial temporal pathway (Kravitz et al. 2011b). It may, therefore, not come as a surprise that the human PPA, besides responding to spatial layout (Epstein and Kanwisher 1998), also displays more activity in response to indoor than outdoor scenes (Henderson et al. 2006) because indoor scenes encompass more objects. Similarly, even a room with a single object elicits different activation patterns to the same room without any objects (Harel et al. 2012; see also, MacEvoy and Epstein 2011).

Additional evidence for multiple functions of the PPA comes from a connectivity study by Baldassano et al. (2013) who demonstrated that the PPA is not a homogeneous area. The anterior part is coupled with the RSC and the caudal inferior parietal lobe (cIPL), which in turn are connected to the anterior hippocampus (Aminoff and Tarr 2015; Baldassano et al. 2016). This indicates that memory and navigational properties are restricted to the anterior part. In contrast, the posterior PPA was connected to occipital visual regions (LOC and OPA) and was, therefore, responsible for objects within scenes. A developmental study on scene perception by Chai (2010) confirms the involvement of the PPA in the memory processes. The posterior part of the parahippocampal gyrus (PHG), which also encompasses the PPA, was the most relevant area for successful categorization and remembering of high-complexity scenes that feature numerous objects. Unfortunately, this study did not specifically localize the PPA and its anterior and posterior parts. Our study provides additional indication that the more anterior PPA voxels may be more sensitive to functional properties between objects in the scene, whereas the posterior counterparts seem to be more responsible for layout properties. Although the direction of the correlation between the anterior–posterior voxels and their categorization success was always consistent with this conclusion, the correlation itself was rather low.

The right PPA was more sensitive to both layout and, in particular, randomization than the left PPA (Fig. 3). This is a somewhat surprising result given that in some of the previous studies no big differences were found and the statistics on both PPA were often collapsed into a single ROI (Epstein and Kanwisher 1998; e.g., Harel et al. 2012; Bastin et al. 2013). On the other hand, studies that manipulated relations of objects (Aminoff et al. 2007; Bar et al. 2008; Bilalić et al. 2010, 2012) found more pronounced right PPA activation, although none of these studies explicitly tested for differences as we did here. It is possible that the right PPA’s sensitivity to object relations and layout is related to its more form-specific analyses in visual scene processing (Walther et al. 2009, 2011). The left PPA, on the other hand, seems to be more specialized for form-abstract conceptual processes that involve conceptual but not visual similarities in scenes (Stevens et al. 2011). The exact difference in the function of the left and right PPAs may be one of the questions for future studies.

Just like the right PPA, the left and right RSC seem to parse scenes in such a manner that both the layout properties of scene and the objects within it are processed together (see also Harel et al. 2012) because both the left and right RSC were sensitive to both factors (see Figs. 2, 4). This was confirmed as early as the univariate analysis and the same pattern was also found with MVPA. However, the cross-categorization enabled us to go beyond this as it established that the RSC only used the information about the object relations and not the layout for the differentiation of new stimuli (see Fig. 3b). One explanation for this pattern of results is that objects and their relations may be more relevant for navigation within the scene environment (Epstein et al. 2007; Bastin et al. 2013; Aminoff et al. 2013) than the spatial layout, at least in this particular task. Unlike most previous research, our paradigm forced the participants to actively manipulate the stimuli, which requires knowledge retrieval about the object relations from long-term memory (Bilalić et al. 2010, 2012; Võ and Wolfe 2013b). Quickly grasping the environment through, among other processes, recognizing typical objects and the relations between them, is essential for successful navigation within the environment.

The navigation that is necessary in our search paradigm may be one of the reasons why the OPA (see Appendix for detailed results) was relatively successful in differentiating between normal and random rooms. Nevertheless, only one such comparison was significant (e.g., normal versus random rooms without layout in the left OPA, see Fig. 5). On the other hand, none of the layout comparisons was reliably successful. The role of the OPA in our paradigm is currently unclear and more research may be needed to understand its role in parsing object relations and spatial layout of rooms.

Our current study emphasizes a difference between paradigms that involve passive watching of scenes and active use of domain-specific knowledge. The prevailing opinion in the fMRI community seems to be that processes related to scene perception are automatic and will, therefore, become apparent even in paradigms where participants passively observe scenes (e.g., Harel 2015a). This is reflected in the choice of the paradigms that feature passive observation of stimuli, a particularly convenient way for conducting studies in MRI scanners. However, the role of attention in scene perception is far from resolved, as one can observe in various debates on the same or similar issues outside the fMRI community (e.g., Gronau and Izoutcheev 2017). Our study adds to the current debate using a paradigm that involves active use of domain-specific knowledge. More importantly, our study illustrates once again that other factors also influence activations in various brain areas, such as the PPA and RSC. These factors may require attention, but may not be its direct product. Attention is seen as the most commonly used confounding factor in fMRI studies (Esterman and Yantis 2009). However, if it were only attentional effects driving the result in our study, we would most likely see them in the control ROIs, the right LOC and the right FFA, which are well known to react to attention and expectation (Summerfield et al. 2008; Summerfield and Egner 2009). Given that this is not the case, it seems rather that they may well elicit additional processes related to domain-specific knowledge that are not automatically stimulated in classical observational paradigms (Harel et al. 2010, 2014).

Besides the active participation of the participants discussed above, one of the possible reasons why we managed to demonstrate the right PPA’s sensitivity to object relations, something that proved elusive in previous studies (Epstein and Kanwisher 1998; Park et al. 2011; Kravitz et al. 2011a; Harel et al. 2012), is the randomization paradigm. By directly manipulating the meaningfulness of the object relations within scenes and combining it with a more sensitive MVPA (Haynes and Rees 2006; Norman et al. 2006), we uncovered that the right PPA was sensitive to object relations. One could argue that scattering objects in the room randomly nevertheless changes the global layout of the stimuli and, therefore, not only disturbs the relations between those objects, but also influences the spatial layout of the scene. This is certainly a possibility, especially in other outdoor categories of scenes. The rooms, however, have walls as typical constraints that make most of the global space. This is evident by the stream of research that has contrasted empty rooms with walls against the same rooms with objects (Epstein and Kanwisher 1998; Epstein et al. 1999; Harel et al. 2012; Bettencourt and Xu 2013; Kamps et al. 2016), as well as rooms with and without walls to investigate spatial layout (Epstein and Kanwisher 1998; Harel et al. 2012; Kamps et al. 2016).

Despite the demonstrated advantages of the randomization paradigm, there are also drawbacks. As a consequence of using multi-object stimuli typical of real life, their randomization produces unnatural stimuli. Our version of randomization avoids unnatural positioning of objects (e.g., floating in the air) but introduces another problem as objects are consequently placed in their unnatural positions (e.g., a sofa on the ceiling is inevitably upside-down). These differences may, for example, produce fine graded visual differences between conditions. These differences may not be processed in the PPA and RSC but rather in early visual areas, which then forward the information to the high-level visual areas. The sensitivity of the PPA and RSC to the object function may then be a consequence of these differences in visual features and not of sensitivity to object relations. It is impossible to rule out this possibility completely without additional experiments which would control for the confounds in the randomization paradigm. However, the pattern of results in the LOC, the area generally assumed to process visual features of stimuli (Malach et al. 1995; Grill-Spector et al. 1999; Vinberg and Grill-Spector 2008), speaks against this possibility. The LOC was not sensitive to the layout manipulation, which might have been expected from previous research (Epstein et al. 2003; Park et al. 2011; Harel et al. 2012). The same can be said for V1 (see Appendix). The randomization has not produced any noticeable differences in the LOC, even when the more powerful MVPA was used in the analysis.

The other control area, the right FFA, also provides evidence against a strong version of this hypothesis. The FFA is not only sensitive to multi-layered stimuli, which promotes holistic processing (Bilalić et al. 2016; Bilalić 2016), but it also seems to be sensitive to the manipulation of object relations caused by the randomization paradigm, at least with chess stimuli and chess experts (Bilalić et al. 2011; Krawczyk et al. 2011; Bartlett et al. 2013; Righi et al. 2013). As such, it is an ideal area to demonstrate sensitivity to the randomizations of objects within rooms. This is not the case here as the FFA could not differentiate based on either the layout or the object relations in rooms. It is possible that the difference between chess and room stimuli employed in the studies is responsible for the differing patterns of results. It is, however, also possible that the FFA simply does not process scene features necessary for scene perception and orientation, unlike the scene areas PPA and RSC.

Finally, the visual search paradigm inevitably produces different patterns of eye movement, especially when combined with the randomization paradigm. This means that participants were allocating attention differently and perceiving different objects. The obtained effects in the PPA and RSC, in particular on the normal and random rooms, could be then a product of these differing eye movements and consequently differently focused objects. This possibility gains credibility if we consider that different object may elicit different activation in the object-sensitive areas such as LOC. These early visual regions could then forward processed information for further processing to the regions later in the visual streams such as the PPA and RSC.

Although there does not seem to be a direct way to test for this possibility in our design, we believe that this explanation is unlikely in this particular context. The attended objects may be different but the same typical room objects still appear. It is probable that the participants will in both normal and random rooms pay attention to the same or similar objects. If this is not the case, one would expect that the control areas, the LOC and FFA, may also be sensitive to the same effects, at least to an extent. In particular, the LOC, would be expected to pick up these visual differences. LOC has been shown to be sensitive to contextual effects in visual search paradigms (Preston et al. 2013). Similarly, MacAvoy and Epstein (2011) also demonstrated that the LOC activity of the individual objects in isolation (e.g., oven, fridge) can be used to correctly categorize typical scenes that are made up of these objects (e.g., kitchen). However, the LOC could not differentiate between the normal and random rooms in our study. If the regions in the early visual stream do not show sensitivity towards different eye movements and consequently differently attended objects, it is difficult to believe the effects in the later regions, the PPA and RSC, should reflect these differences.

The MacAvoy and Epstein study (2011) offers additional clues that the effects in the PPA and RSC may not be a consequence of possibly differently attended objects. The categorization of scenes based on the combination of the activity of its individual objects seems possible only in the LOC and not the PPA (MacAvoy and Epstein 2011). This may seem like an argument against the PPA processing of object relations. One needs, however, to keep in mind that our study uses the same type of objects—objects typical for rooms. The MacAvoy and Epstein study used different objects (e.g., oven vs. bathtub) to differentiate between different scenes (e.g., kitchen vs. bathroom). The MacAvoy and Epstein study may rule out the PPA’s parsing of semantic relations between objects (e.g., Aminoff et al. 2007; Bar et al. 2008) but it does not rule out the processing of functional and spatial relations between objects in the PPA.

Our stimuli were scenes of living rooms. This raises the question of whether the same findings generalize across other more or less similar stimuli. There are reasons to believe that other indoor environments, such as kitchens and bathrooms, would follow the same pattern of results. They are also made up of typical objects which are placed in a typical functional manner. Placing a washing machine on the wall and the sink on the floor may have similarly disruptive effects to placing a sofa on the ceiling. The situation becomes more complicated when we move outdoors and consider naturalistic scenes such as, for example, the sun over a hill. The elements in these scenes (e.g., hill) are arguably more difficult to manipulate than the room furniture. A more natural manipulation of these scenes would involve rotating the whole scene to a certain degree (e.g., 90°) or even turning it upside-down (Yin 1969). However, this manipulation is arguably more suited for layout properties of the scene than functional and spatial relations of the elements within the scene (Bilalić 2017).

The other question for future studies is whether the effects would be obtained in the common paradigm where the stimuli are passively observed. This would indeed be a test of the widespread assumption that scene processing is at least partly automatic. Given the difficulties the previous studies had in establishing the role of the PPA in object perception in the scenes (Epstein and Kanwisher 1998; Epstein and Ward 2010; Kravitz et al. 2011a; Harel et al. 2012), one can assume that the passive observation would not produce the same effects as in this study which employed active visual search. The possible no-difference results in the passive observing paradigm is ultimately weak evidence for the role of the PPA in parsing object functions. That is why we believe that the active search paradigm employed in this study, is the better way of understanding the PPA’s role in the perception of objects within scenes.

Overall, our study does not only underline the value of the randomization paradigm that has been making a comeback in visual scene research (Võ and Wolfe 2013a, b), but also the importance of employing paradigms that require active participation and retrieval of domain-specific knowledge (Bilalić et al. 2010, 2011, 2012; Harel et al. 2010, 2014). These paradigms, such as the visual search employed here, have long been used in behavioral research and present an important alternative as well as a way to supplement the common paradigms that rely on passive observation and automatic retrieval.

## Appendix

### Univariate fMRI analysis

Figure 4 shows the neural responses in the left and right PPA and RSC when the participants were actively looking for an object in rooms. We first present the results of 2 × 2 ANOVA with the main factors of randomization (normal and random rooms) and layout (rooms with and without walls) for each single ROI. We then include the side of the ROI (left and right) as the third factor. For the left PPA, the neural activity was the same irrespective of the object arrangement in the rooms [main effect of randomization: *F*(1, 14) = 0.1, *p* = 0.99] and it did not matter if the room were with or without layout [interaction randomization × layout: *F*(1, 14) = 1, *p* = 0.34]. However, the presence of layout modulated the neural response as the rooms with layout elicited stronger activation than the rooms without layout [main effect of layout: *F*(1, 14) = 48.5, *p* < 0.001]. The same pattern of results was observed for the right PPA, although here the randomization was almost significant [*F*(1, 14) = 4.3, *p* = 0.06]. Again, the rooms with layout elicited higher activation [*F*(1, 14) = 96.4, *p* < 0.001], whereas there was no interaction between layout and randomization [*F*(1, 14) = 0.4, *p* = 0.53]. There were no differences between the left and right PPA (2 × 2 × 2 ANOVA between layout × randomization × side did not produce significant results—all *F*s < 1).

In contrast to the PPA, both RSC regions of interest showed a different pattern of activation. The left RSC was more activated in normal rooms than in random ones [main effect of randomization: *F*(1, 14) = 7.2, *p* = 0.018] and was also more activated in rooms with layout than without layout [*F*(1, 14) = 44.3, *p* < 0.001]. The pattern was consistent across both factors as there was no interaction [*F*(1, 14) = 1.2, *p* = 0.29]. The right RSC displayed the same pattern of activations: more activation in normal than randomly arranged rooms [*F*(1, 14) = 6.4, *p* = 0.025], more activation in rooms with layout [*F*(1, 14) = 41.1, *p* < 0.001], and no interaction between the two factors [*F*(1, 14) = 2.5, *p* = 0.14]. The right RSC was more activated than the left RSC [main effect of side: *F*(1, 14) = 8.8, *p* = 0.011] and the rooms with layout activated more the right RSC than the left RSC [side × layout interaction: *F*(1, 14) = 12.9, *p* = 0.003].

The left OPA was not responding differently to normal and random room [main effect of randomization: *F*(1, 14) = 1.9, *p* = 0.19]. The same absence of differences was also found between rooms with layout and without [main effect of layout: *F*(1, 14) = 0.8, *p* = 0.78]. The layout and randomization were not engaging the left OPA [interaction: *F*(1, 14) = 0.2, *p* = 0.89]. The same pattern of results was found in the right OPA: main effect of randomization was not significant [*F*(1, 14) = 0.4, *p* = 0.56] as well as main effect of layout [*F*(1, 14) = 1.5, *p* = 0.24] and interaction [*F*(1, 14) = 0.8, *p* = 0.78]. There were also no differences between the left and right OPA (2 × 2 × 2 ANOVA between layout × randomization × side did not produce significant results—all *F*s < 1).

Unlike the PPA and RSC, the right LOC did not show any effects—see Fig. 5. Neither did randomization [*F*(1, 14) = 0.1, *p* = 0.80] or the layout [*F*(1, 14) = 0.1, *p* = 0.72] played a role. There was also no interaction between the two factors [*F*(1, 14) = 0.2, *p* = 0.63]. The same lack of effects was found in the control ROI, the right FFA. The main effects of randomization [*F*(1, 14) = 0.1, *p* = 0.83] and layout [*F*(1, 14) = 1.9, *p* = 0.19] were not significant, and there was no interaction between the two factors [*F*(1, 14) = 0.1, *p* = 0.71]. Similarly, there were no effects evident in right V1 [main effect of randomization: *F*(1, 14) = 0.3, *p* = 0.85; main effect of layout: *F*(1, 14) = 1.7, *p* = 0.22; interaction: *F*(1, 14) = 1.8, *p* = 0.20].

### Multivariate analyses of OPA

Similar to other scene areas, the OPA is reacting to scene more than to other stimuli (Bettencourt and Xu 2013). The OPA is also more activated when rooms with furniture are shown than when the same furniture is presented without the room confines (Bettencourt and Xu 2013). Recent studies suggest that the OPA is important for recognition of landmarks through their visual features (Marchette et al. 2015), as well as for identifying potential paths in a scene (Bonner and Epstein 2017). It is currently unclear whether the OPA reacts and to what extent to the spatial layout and object placements.

As can be seen in Fig. 6, the left OPA did not quite reach the significance level (*p* < 0.0125 after correction for multiple comparisons) for discriminating between normal rooms and random rooms with layout [*t*(14) = 2.48, *p *= 0.014], but did reach the significance level for differentiation between normal and random rooms without layout [*t*(14) = 3.29, *p *= 0.003]. The left OPA did not, however, differentiate between rooms with and without layout—*t*(14) = 0.01, *p *= 0.99 for normal with vs. normal without layout, and *t*(14) = 0.61, *p *= 0.55 for random with vs. random without layout rooms. We found the same tendency for right OPA, but the randomization comparisons did not quite reach the significance level [normal versus random rooms with layout: *t*(14) = 1.59, *p *= 0.066; normal versus random rooms without layout: *t*(14) = 2.32, *p *= 0.018]. There were no significant differences for the layout comparisons [normal rooms with layout versus normal rooms without layout: *t*(14) = 0.27, *p *= 0.40; random rooms with layout versus random rooms without layout: *t*(14) = 1.29, *p *= 0.11]. The direct comparison between the left and right OPA did not result in any significant effects (all *F*s < 1, ns.). Similarly, there was no significant correlation between the MVPA accuracy in the OPAs and the reaction time of participants (all *r *< 0.23).

Figure 7 shows that the left and right OPA could not successfully cross-categorize rooms across either randomization or layout factor (all *p* > 0.10).

### Multivariate analyses of V1

Unlike the previous ROIs, the right V1 was the anatomically defined V1 which was standard for all individuals (for more details, see the Method section of the main text). The analyses were then made on the normalized unsmoothed functional scans, which may explain the inconsistent results. Figure 8 shows that the V1 could reliably differentiate only between normal and random rooms without layout [*t*(14) = 5.21, *p *< 0.001]. All other comparisons were not significant (*p* > 0.10). Similarly, the cross-categorization procedure was not reliable in the V1 (*p* > 0.10).

## Footnotes

1 The studies included attentional control (e.g., detection of repeated stimuli) but no actual domain-specific manipulation with stimuli.

2 One should note that our rooms did not have texture on the walls. Recent research (Lowe et al. 2016) demonstrates that the PPA is sensitive to texture and future research should take this finding into account when designing room stimuli.

3 The PPA split into anterior and posterior PPA confirms the trend of the anterior PPA being more responsive to the randomization factor and the posterior PPA to the layout factor, but the differences were not significant. However, we believe that the correlation represents the anterior–posterior division better than a simple division of the PPA into the anterior and posterior part because it captures the whole spectrum of the data along the anterior–posterior axis.
